# The Present Status of Conservatism in the Surgical Treatment of Tubes and Ovaries

**Published:** 1908-05

**Authors:** John Egerton Cannaday

**Affiliations:** Surgeon-in-Charge, Sheltering Arms Hospital, Hansford, W. Va.


### ABSTRACTS.

### The Present Status of Conservatism in the Surgical Treatment of Tubes and Ovaries.

By JOHN EGERTON CANNADAY, M. D.
Surgeon-in-Charge, Sheltering Arms Hospital, Hansford, W. Va.

[*Author's Abstract*]

AFTER reviewing the history of attempts at conservatism and quoting the words of the elder Emmet in which he expressed the hope that future generations of women might be allowed to go uncastrated the author says: We all know that even the least of the atoms has its definite value and its purpose to fulfil in the harmonious whole of natural plan. We have been long taught that when a limb was injured, a hand mangled, or an abscess formed, to make every effort to save the part. Until rather recent years these saving methods did not seem to apply to the pelvic organs of women at all.

He addressed circular letters of inquiry to 35 of the leading gynecologists of America, in regard to their position in the matter of ovarian and tubal ablation. The answers received would indicate that at least half our gynecologists are very conservative. A number of others practice palliative treatment and do without operation except when indications are urgent, then being most radical, while a smaller number evidently operate early and often. The technic of plastic repair work is described. Chromicised catgut is generally used as a suture material. Conservative work is not attempted in the presence of pus. The author believes that, barring the presence of the menopause, inflammation, pus, tuberculosis and malignant disease, conservative work should be done; that every organ or part of an organ consistent with the health and well being of the patient should remain undisturbed; that in these cases there is much room for exercise of good judgment and due discrimination; that the risks of infection and of secondary operation from portions left behind are rather remote in properly selected cases.

He summarises as follows: The majority of the gynecologists interrogated favor a restricted conservatism; that the number of pregnancies occurring after tubal operations is very small; that the results after plastic work on the ovaries is better, that age, the presence of pus, tuberculosis and malignant disease indicate as a rule, radical work; that prolapsed ovaries, generally speaking, should be elevated in the pelvis by suspension operations on the uterus, by shortening the ovarian ligament or by placing the ovary in front and on top of the broad ligament; that the functions of the tube and ovary should be preserved whenever consistent with health; that the artificial induction of the menopause brings a very serious disturbance into the life of the patient, and that ovarian transplantation experimentally and clinically has, in a limited field, been productive of satisfactory results.

